# Removal Forces of a Helical Microwire Structure Electrode

**DOI:** 10.3390/bioengineering11060611

**Published:** 2024-06-13

**Authors:** Amelia Howe, Zhanda Chen, Kyle Golobish, Victoria R. Miduri, Derrick Liu, David Valencia, Morgan McGaughey, Emily Szabo, Manfred Franke, Stephan Nieuwoudt

**Affiliations:** Neuronoff, Inc., Cleveland, OH 44106, USA; amy@neuronoff.com (A.H.);

**Keywords:** implantable medical device, tissue encapsulation, device explantation, biomechanics

## Abstract

(1) Background: Medical devices, especially neuromodulation devices, are often explanted for a variety of reasons. The removal process imparts significant forces on these devices, which may result in device fracture and tissue trauma. We hypothesized that a device’s form factor interfacing with tissue is a major driver of the force required to remove a device, and we isolated helical and linear electrode structures as a means to study atraumatic removal. (2) Methods: Ductile linear and helical microwire structure electrodes were fabricated from either Gold (Au) or Platinum–Iridium (Pt-Ir, 90-10). Removal forces were captured from synthetic gel models and following chronic implantation in rodent and porcine models. Devices were fully implanted in the animal models, requiring a small incision (<10 mm) and removal via tissue forceps. (3) Results: Helical devices were shown to result in significantly lower maximal removal forces in both synthetic gel and rodent studies compared to their linear counterparts. Chronically (1 yr.), the maximal removal force of helical devices remained under 7.30 N, for which the Platinum–Iridium device’s tensile failure force was 32.90 ± 2.09 N, resulting in a safety factor of 4.50. (4) Conclusions: An open-core helical structure that can freely elongate was shown to result in reduced removal forces both acutely and chronically.

## 1. Introduction

The removal or explant of medical devices is a crucial consideration throughout the entire product life cycle, and the ease of device removal is a concern for both clinicians and patients [1,2]. Neurostimulators and other neuromodulation devices are commonly removed due to various hardware-related or biological complications. It has been reported that up to 38% of this class of medical devices required surgery to resolve complications related to the devices, which necessitated device removal [3]. While traditional removal methods require invasive surgery, the in-office removal of neuromodulation leads with local anesthesia has recently been shown to be safe, effective, and well tolerated [4]. The removal of these implanted devices may also be required in situations such as before a patient undergoes magnetic resonance imaging (MRI), at the end of a trial device period [5], or due to insufficient pain relief [6].

Despite its significance, the removal of neuromodulation leads is not widely reported. To the authors’ knowledge, multiple case reports and letters to the editor have been used to underscore clinical phenomena associated with the removal process [7,8,9,10,11,12,13]. These studies discuss procedural safety and efficacy but do not characterize the mechanisms, mechanical forces, or conditions associated with the device removal process in which local tissue invariably experiences trauma. The mechanical forces experienced by the device and tissue throughout lead removal must be studied to understand, predict, and minimize the prevalence of device fractures. Such an understanding would drive future design considerations for medical devices, such as form factor and the refinement of the removal procedure itself. Lead fracture during removal (a consequence of the removal force exceeding the tensile strength) can also preclude patients from undergoing an MRI, as the presence of metallic fragments in vivo can result in image artifacts, tissue heating, or other adverse magnetic field interactions under certain imaging conditions [14].

Clinically implemented neurostimulators, including cardiac pacemaker leads, Spinal Cord Stimulation (SCS) leads, and Peripheral Nerve Stimulation (PNS) leads, have adopted linear designs with respect to the direct tissue contacting interface [15]. Linear leads may either be ring leads, which are composed of multiple conductive rings on a coated cylindrical body with wire conduits attached to each of the stimulating ring contacts, or they may include variations on “paddle” lead configurations, such as those used in SCS and cortical applications [16,17]. In structural contrast to these linear configurations, helical lead configurations have been used for some peripheral nerve cuffs [15,18], and percutaneous externalized leads with helical structures have been employed for peripheral neurostimulation use [19,20]. As an additional benefit, the helical (coiled) structure of these devices has also been shown to reduce infection rates at the site where the device crosses the skin [21].

In this study, we aimed to quantify the differences between the removal forces of a neurostimulation lead when arranged in a linear versus a helical design configuration. The removal forces of these two structural arrangements were assessed both on the benchtop in synthetic gel models and following chronic implantation in animal models. Specifically lacking in the literature are data on the mechanics of the removal of chronically implanted and removed neurostimulators. As such, we chronically implanted and collected removal force data from both small and large animal models for the helical device configuration. These results will drive the design of an injectable electrode platform with a focus on removal safety.

## 2. Materials and Methods

**Figure 1:**
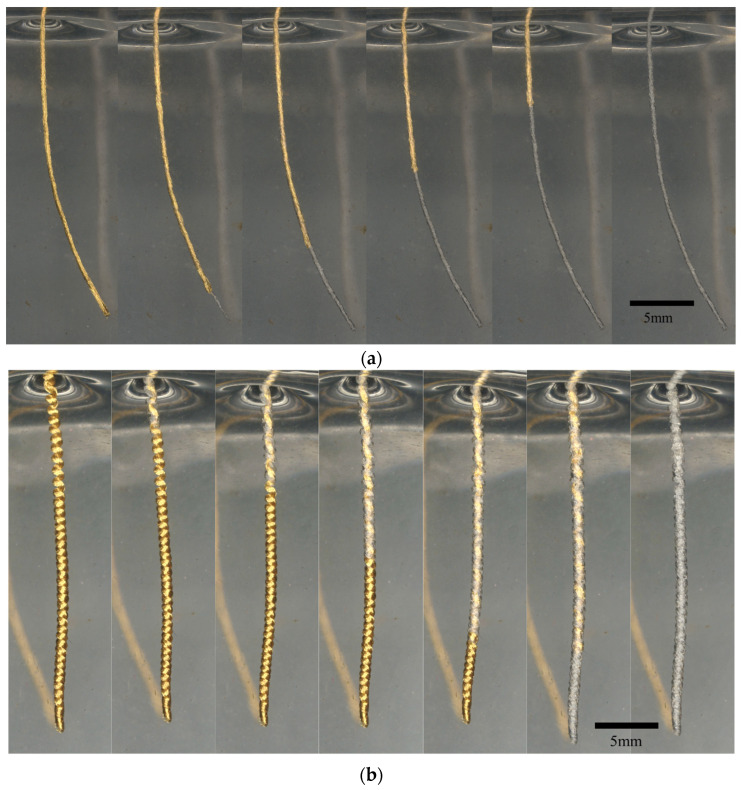
The removal of a (**a**) linear and (**b**) helical Gold microwire device from a synthetic gel model with sequential images from left to right. The scale bar is 5 mm.

Linear (Figure 1a) and helical (Figure 1b) structure devices were manufactured from 25-micron Gold (Au, 99.99%, Heraeus, Hanau, Germany) or Platinum–Iridium (Pt-Ir, 90-10, Prince and Izant, Cleveland, OH, USA) microwires with or without polyolefin heat shrink tubing (low-density polyethylene, Lubrizol, Wickliffe, OH, USA). The coating was included on devices used for electrical stimulation studies, which is outside the scope of the data presented here. Linear and helical devices were manufactured as 100 parallel strands of microwire with a 2.8–3.2 mm per turn twist with a nominal diameter of approximately 280 microns. To impart a helical structure, the linear devices were wound around a 0.25 mm mandrel, which resulted in a tightly packed (lowest pitch with minimal inter-coil spacing) helical structure with an open core. Coating was applied to devices after twisting but prior to winding. In preclinical models, these devices were injected with an 18-gauge needle and 1 mm plunger-based delivery system. Devices of various lengths were prepared for benchtop and animal studies, all of which are noted specifically in Section 3.

All studies involving vertebrates (Rodent and Porcine) were approved by the Case Western Reserve University Institutional Animal Care and Use Committee (CWRU IACUC). All research animals were housed, fed, and cared for in strict accordance with the National Institutes of Health Guide for the Care and Use of Laboratory Animals. The specific animal models used in the studies presented here included Sprague Dawley rats, Yorkshire farm pigs, and Yucatan minipigs.

### 2.1. Imaging

This study used microscopy, fluoroscopic, and photographic imaging methods. Microscopy images of the devices were taken on a benchtop microscope (Tomlov, Shenzhen, China) or a high-content imager (KEYENCE VHX-7000, Itasca, IL, USA) before, during, and after removal. Fluoroscopic images were taken using a Philips Veradius system (Amsterdam, The Netherlands) to locate devices for removal, to record the removal process, and to ensure the complete removal of devices while assessing device fracture. Photographic images were edited for cropping, white balancing, and hue adjustments with the publicly available GNU Image Manipulation Program (version 2.10.36).

### 2.2. Tensile Testing

**Figure 2:**
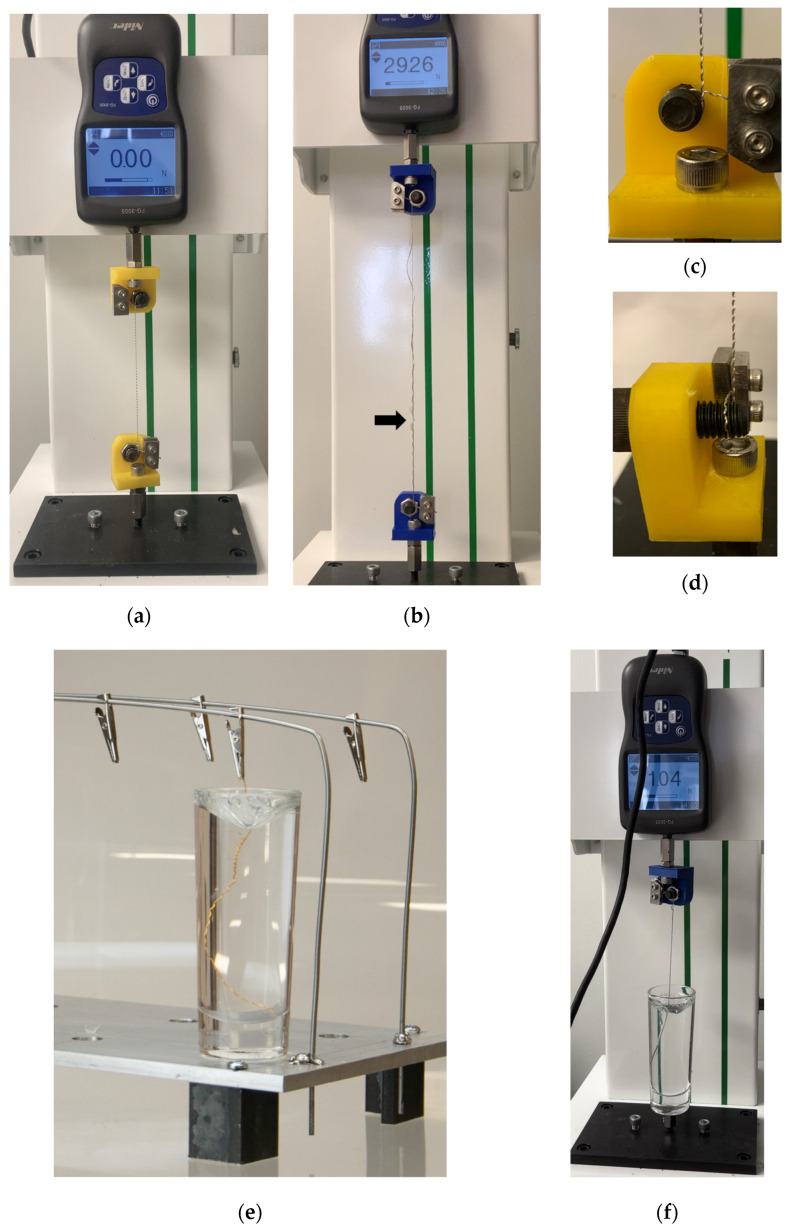
Benchtop tensile force testing equipment. Vertical test stand used for tensile testing with force transducer prior to loading (**a**) and at moment of tensile failure (**b**) with arrow pointing to failure site. Custom capstan grips (**c**,**d**) that were used in all testing increased test accuracy. For synthetic gel removal tests, (**e**) devices were suspended in gel prior to reheating in vacuum oven. (**f**) Same custom capstan grips were also used in tensile removal testing of gel-embedded devices.

To measure the uniaxial tensile strength of the devices, a mechanical test stand (Nidec Corporation, Kyoto, Japan) was used (Figure 2a). The tensile load was applied at a standard rate of 25 mm/min until failure on all devices (Figure 2b). Custom capstan grips were designed to accommodate the small diameter of the device and limit stress concentrations leading to premature fracture outside the gauge region (Figure 2c). These grips distribute part of the tensile load around the diameter of the sheave, and the remainder of the load is applied at a clamp (Figure 2d). To ensure that the design of the grips would sufficiently secure the device, the capstan equation was used to calculate the change in tension due to a flexible rope wrapping around a stationary cylinder under load [22]:

(1) $$T_{Load}=T_{Hold}e^{\mu \Theta }$$

Here, T_Load_ is the tensile force applied to the rope before the sheave, T_Hold_ is the tensile force applied after the sheave, μ is the coefficient of friction, and θ is the angle between the entrance and exit points. The force applied after the sheave, T_Hold_, was less than the force required to break the clamped device. The maximal failure force for any devices was estimated at 40 N (T_Load_) with a target clamping force of 10 N or less (T_Hold_). A low steel–steel static friction coefficient of friction of 0.5 was used [23], as direct values for the used materials were not available. As such, the required angle θ was calculated as 201.1°. For the design of the custom capstan grips, this angle was increased to 250.0° as a precaution, which allowed us to define the number of rotations the specimen needed to complete. The radius of the sheave was required to be sufficiently high so as to not induce a stress concentration while minimizing the length of specimen utilized by the grips. A 4 mm radius for the sheave satisfied both of these considerations, and the use of a threaded bolt additionally provided guides which ensured there was no physical overlap in the test samples.

### 2.3. Synthetic Tissue Model

For benchtop testing, we used Gelatin #0 (Humimic Medical, Greenville, SC, USA), which has an equivalent stiffness and density to muscle tissue as stated by the manufacturer. Gels were heated to 132 °C to bring the material to a liquid state prior to the embedding of devices. Devices were suspended and held in place (Figure 2e) and reheated to the liquid state in a vacuum oven (BOV-50, BEING Scientific Inc., West Covina, CA, USA), after which test articles were left to solidify at room temperature for 24 h. Removal force from the gel was measured for both helical and linear devices utilizing the aforementioned vertical test stand and force transducer (Figure 2f). The lengths of the embedded portion within the gels were varied in order to evaluate length-based variance equivalent to various peripheral nerve depths [24] and compare any differences observed between the linear and helical devices. These lengths are noted in the results and varied from 1 to 8 cm. Devices were removed at a rate of 162 mm/min, which was set at 10% less than the maximal speed of the test frame to ensure the maximum rate was not exceeded during testing.

### 2.4. Rodent Removal Model

Helical and linear devices were implanted in rodents with one end placed on the sciatic nerve and the other end placed under the skin near the ribcage in a caudal to cranial path with the devices crossing over the hip joint and the femur and traversing the biceps femoris muscle. Devices were fully removed from rodents at varying time intervals over a year of implantation with no more than 2 devices implanted per animal bilaterally. During removal, a standard 5 mm surgical blade was used with tissue forceps and a hemostat to locate and extract the proximal (cranial) end of each device. A force transducer (Nidec Corporation, Kyoto, Japan) was attached to the hemostats to collect removal forces.

Following device removals, fluoroscopic images were taken of the removal site to determine whether any fragments remained or if any fractures had occurred. To overcome the imaging resolution limitations of fluoroscopy, Inductively Coupled Plasma Mass Spectrometry (ICP-MS) was performed to measure trace levels of Platinum and Iridium (Plasma Chemistry Laboratory at the Center for Applied Isotope Studies, University of Georgia, GA, USA). Briefly, tissue samples were excised surrounding the removal tract (as marked prior to device removal under fluoroscopy) and trimmed to a weight of approximately 0.75 g. Tissue samples were then processed (wash/dry/freeze-dry/grind/ash) prior to acid digestion and ICP-MS analysis.

### 2.5. Porcine Removal Model

Based on the initial findings in rodents, solely helical devices fabricated from Pt-Ir were selected for implantation in porcine models. Devices were implanted near the porcine tibial nerve, with up to 3 devices per nerve and 6 devices per animal. Devices were then removed at various time points throughout 56 days of implantation using the same force recording process as performed in rodents, albeit with less ability to achieve a pulling force normal to the device path. All procedures were performed in the Surgical Training and Research Core of Case Western Reserve University (STAR Core, Cleveland, OH, USA). During removal, we observed an initial peak and rapid drop (more than 2 N in 0.1 s) in force generated on some samples. Upon further investigation, we determined this initial peak to be the tissue ripping force produced by the surgical hemostats that were clamped directly onto both the tissue and the device itself. When careful dissection was performed to ensure only the device was engaged in the hemostats, the tissue ripping force was not observed. Empirical testing of the tissue ripping force in porcine tissue showed that this force was on average 12.89 ± 3.70 N (n = 5). As such, we excluded this peak from our maximal force calculations whenever present.

### 2.6. Histology

Rodent and porcine tissue samples were collected from multiple regions along device implant paths at terminal dates (post-euthanasia). Devices were implanted in rodent and porcine models to account for the chronic effects of the reactive tissue response to the implant. Histological samples prepared from tissues from which devices were removed proved inconclusive, as locating the removal tract proved difficult. Instead, devices were left intact in the tissue for histology. Samples were excised, trimmed, and immediately placed in a solution of neutral buffered 10% formaldehyde. Samples were formaldehyde-fixed for 7–14 days to ensure formalin penetration into tissue (1 mm fixative penetration per 3 h of fixation). Samples were swapped with an appropriate quantity of 70% ethanol or 70% isopropanol reagent in a 4 °C refrigerator to achieve tissue drying. Tissues were stored in alcohol reagent until shipment. Plastic embedding, sectioning, grinding, and staining were performed by an independent service (Structure, Composition and Histology Core, University of Michigan, Ann Arbor, MI, USA) with expertise in plastic sectioning of metal implants. Stained slides (Toluene Blue or Hematoxylin & Eosin) were scanned using a high-content imager (KEYENCE VHX-7000, Itasca, IL, USA). Physical slides were transferred to an independent histopathologist (Nicholas P. Ziats, Ph.D, Case Western Reserve University, Cleveland, OH, USA) for blinded histopathological analysis.

### 2.7. Statistical Analysis

All statistical analyses were performed with R (version 4.2.3). The Shapiro–Wilk test was performed to evaluate normality. Equal variance was not assumed for any dataset, and a statistical analysis was not performed on sample sizes that were deemed small (n = 3 or fewer). Unpaired two-sample *t*-tests were performed to compare data between groups. Mean ± standard deviation were reported, and sample sizes were noted throughout. In all comparisons, *p* ≤ 0.05 was deemed significant.

## 3. Results

### 3.1. Benchtop Characterization of Removal Forces

Benchtop tensile failure testing was first performed on both Gold and Platinum–Iridium devices to establish a baseline. The tensile failure state for both linear and helical devices is equivalent, as helical devices are first elongated, which returns them to their linear structure, at which point mechanical tensile failure will occur under strain. Platinum–Iridium devices had a tensile failure force of 32.90 ± 2.09 N (n = 26) and Gold devices had a tensile failure force of 4.3 ± 0.5 N (n = 6). Of interest was the effect that chronic implantation in an animal model would have on the ultimate tensile failure force. Following implantation in rodents for 60 days, the tensile failure force of the devices was not significantly impacted for Gold devices (3.9 ± 0.6 N, n = 6, *p* = 0.24).

**Figure 3:**
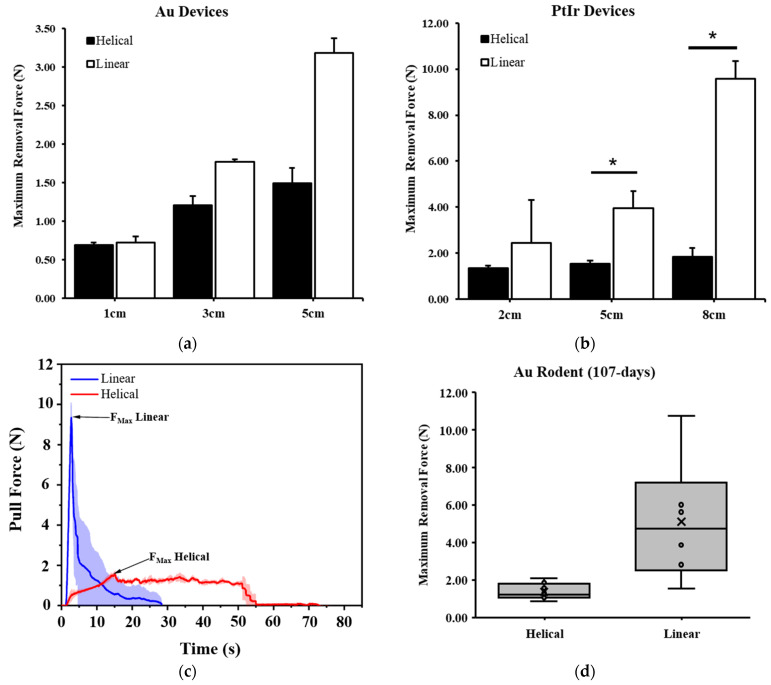
Removal forces of helical versus linear devices for (**a**) Gold devices from synthetic gel in pilot study and (**b**) Platinum–Iridium-based devices at varying lengths, * *p* < 0.01. (**c**) Average cumulative distribution plots of pull force data taken for helical vs. linear samples at 8 cm embedded length. (**d**) Gold helical and linear devices following chronic implantation in rodent model of 107 days.

Gold linear (Figure 1a) and helical (Figure 1b) devices were first evaluated in a pilot test (n = 3 each) in a synthetic gel model, which showed higher removal forces required for longer device lengths (Figure 3a). The experiment was then repeated for Platinum–Iridium (n = 3 to n = 6) devices with lengths extended to 2, 5, and 8 cm embedded depths, each with 2 cm of lead additionally exposed above the gel for mechanical interfacing. Statistically significant differences were observed at 5 cm (*p* = 0.006) and 8 cm (*p* < 0.001). For an increase in length across all samples (1, 2, 3, 5, 8 cm), linear device removal forces increased with length at a much greater rate with a linear trendline slope of 1.220 (R^2^ = 0.838) versus a slope of 0.130 (R^2^ = 0.638) for helical devices. No significant differences were observed between helical devices with different embedded lengths. The difference in average force profiles can clearly be seen in Figure 3c, with maximal forces noted, suggesting that there are fundamentally different methods of force transfer between the linear and helical devices on the synthetic gel.

### 3.2. Chronic Removal Forces

A key shortcoming of the benchtop model is that it did not account for the biological impact of tissue ingrowth and adhesion as a standard immune response to the implantation of medical devices. Therefore, due to the formation of adhesions on implanted devices and the heterogeneity of tissue mechanics [25], biological testing was necessary to accurately assess the forces experienced during the removal of linear versus helical devices.

Following 107 days of implantation in rodents, linear and helical Gold devices were removed, and forces were recorded during removal. Seven-centimeter-long gold devices were implanted bilaterally with each device structure in each animal (n = 8). Of the devices implanted, two of the linear devices were self-explanted, whereas all helical devices remained anchored. The maximal force required to remove the linear devices (n = 6) was 3.7 times greater than the maximal force required to remove helical devices (n = 8) at 1.37 ± 0.44 N vs. 5.11 ± 3.24 N, which is a significant finding (*p* = 0.036, Figure 3d).

**Figure 4:**
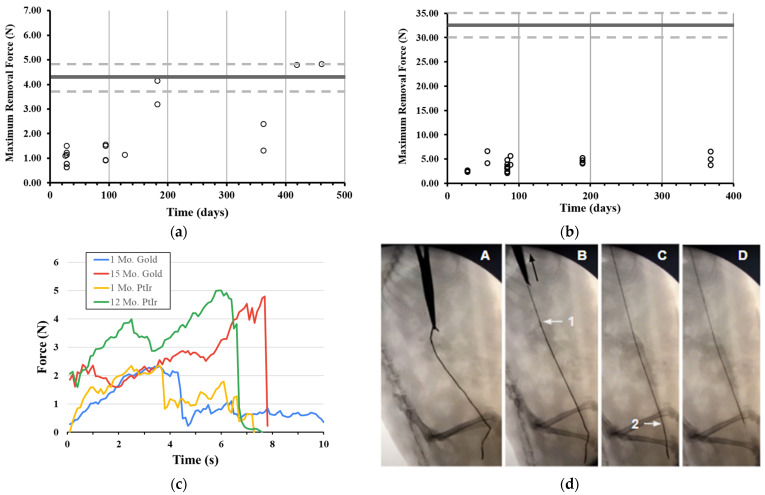
Removal forces of helical devices from rodent model and chronic time points. (**a**) Gold helical microwire devices implanted and removed at various time points across 15 months, with horizontal line representing ultimate failure force of devices (dotted line for standard deviation) and (**b**) same plot for chronically implanted Platinum–Iridium helical devices, with higher ultimate failure force represented by horizontal line. (**c**) Representative force time plots of helical devices during removal. (**d**) Sequence of fluoroscopy images of removal of helical device showing sequential release of device from tissue (arrows 1 and 2 pointing to location where transitions from helical to linear occurs, seen as change in device opacity from black to gray).

We also chronically evaluated the removal force of Gold and Platinum–Iridium devices and pooled the data for different lengths of helical devices as we did not see a significant length dependent effect from our benchtop data. The maximal force of removal of Gold helical devices (4.5, 5, and 7 cm in length) for all time points over a 15 month period (n = 19) is shown in Figure 4a, with the mean ultimate failure force of Gold devices noted with a horizontal line and a dotted line to indicate the standard deviation above and below the mean. Only one Gold device fractured during the removal process.

Chronic removal forces from rodents for Platinum–Iridium helical devices (7 and 10 cm in length) are shown in Figure 4b (n = 26). Over that time period, no removal force exceeded 6.63 N, and the much greater maximal failure force of the Platinum–Iridium devices is also noted with a horizontal line (dotted lines as standard deviation from the mean), showing a greater safety factor for the significantly stronger Platinum–Iridium helical devices compared to the Gold helical devices. Time plots for the removal forces from rodents are overlaid in Figure 4c for various time points and both materials for the chronic implantation studies. Stepwise uncoiling of a Platinum–Iridium device during removal can be seen under fluoroscopy following 60 days of implantation in Figure 4d.

### 3.3. Removal Forces from a Large Animal Model

**Figure 5:**
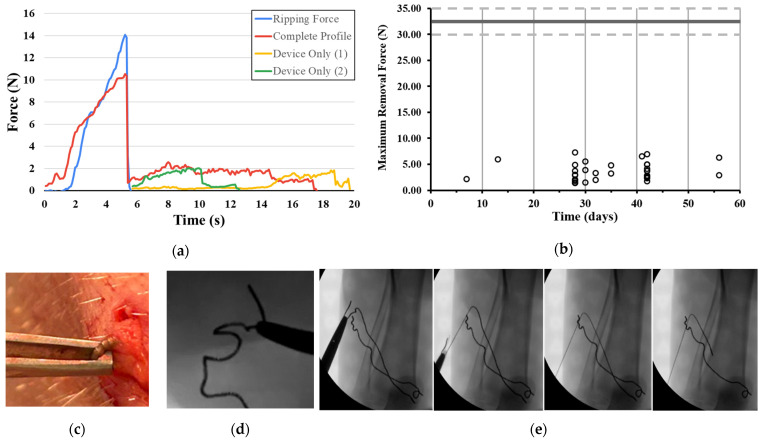
(**a**) The force time plot of different removal force profiles detected during the explant of helical devices from a porcine model, highlighting the abrupt transition from tissue ripping force to true removal forces imparted on the device during pull-out. (**b**) The maximal removal forces measured across 56 days for helical Platinum–Iridium devices removed from the porcine model, with a horizontal line indicating the ultimate failure force of the device (dotted lines for standard deviation from the mean). (**c**,**d**) Images of tissue forceps engaging with a helical device prior to removal and fluoroscopy. (**e**) Sequential fluoroscopy images of the removal of an implanted helical device from the hindlimb in the porcine model (day 42) next to another implanted device.

Since rodent animal models do not share the same anatomical scale as human patients, removal force characterization was repeated in porcine animal models. As such, we injected helical Platinum–Iridium devices (all 10 cm in length) onto the tibial nerve and near the lumbar dorsal root ganglia in farm pigs and minipigs. Multiple devices (up to 8) were placed in each animal, with a subset removed to quantify forces in the more biologically relevant model. The representative time force plots overlaid for tissue ripping and subsequent device removal are shown in Figure 5a, again showing the drastically greater force required to rip tissue with respect to the removal of an implanted helical device. Across a period of 56 days, devices were removed from pigs, for which the maximal removal force never exceeded 7.30 N, as shown in Figure 5b (n = 34). No adverse effects were reported following the implantation or removal of devices, and no devices fractured during the duration of implantation or during the removal procedure.

Similarly to the rodent procedures, removal was achieved with a small incision aligned under fluoroscopy over the device and additional microdissection to free the device from surrounding tissue, allowing for full engagement of the device with hemostats, as shown in Figure 5c,d. The representative fluoroscopy of one of two implanted devices being removed from the tibial nerve is shown in Figure 5e. Despite best efforts to remove the devices at an angle normal to the lengthwise orientations of the devices, space and anatomical limitations resulted in non-normal pulling forces in the animal studies (Figure 5e). This non-normal application of force may result in a potential overestimation of the pulling force where the removal force vector is not parallel with non-parallel forces, such as adhesion or friction [26].

### 3.4. Tissue Response

**Figure 6:**
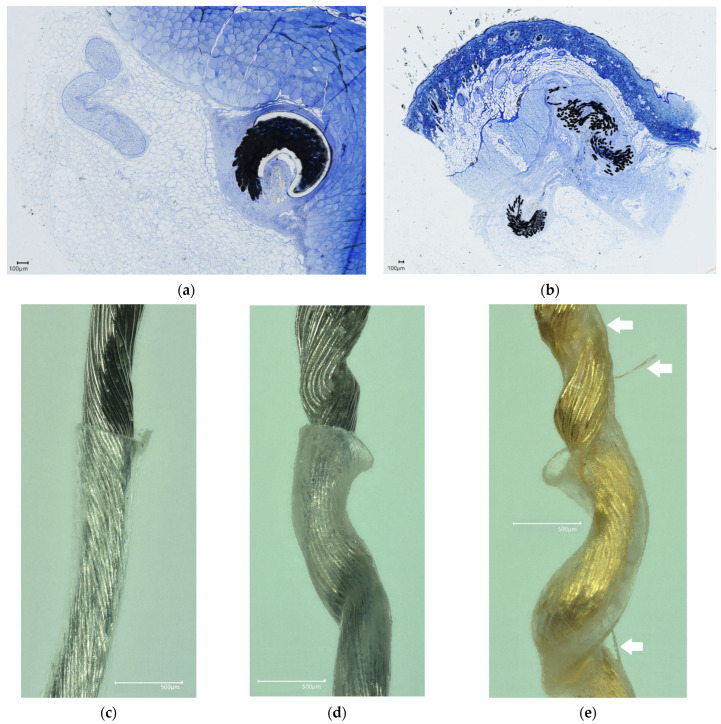
Histological sections with Toluene blue staining of implanted devices and visual assessment of removed devices. (**a**) Histology of electrode on nerve and tissue ingrowth between microwires at transition point from coating to no coating at 5 months in rodents. (**b**) Histology of subcutaneous component of device in pig following 1 month of implantation. Scale bars are 100 microns. Microscopy image of coating to no-coating transition point of (**c**) control non-implanted Platinum–Iridium device, (**d**) Platinum–Iridium device implanted for 12 months, and (**e**) Gold device implanted for 15 months with arrows pointing out tissue extracted with device during removal. Scale bars are 500 microns.

Shown here are images of the stained slides prepared from a rodent implant of 5 months (Platinum–Iridium helical device) at the site placed on the nerve target (tibial nerve) (Figure 6a) and a porcine implant of 35 days of the same device type placed under the skin (Figure 6b). The Toluene blue stain shows the approximate border of the tissue encapsulation, with a presence of tissue ingrowth between the microwires of the implanted helical devices. The histology of the linear devices was not prepared, and as shown here, a cross section of the helical device represents a linear section of the device, although curved. Independent analyses of stained slides by a histopathologist noted a thin capsule adjacent to the implant (25 to 75 microns), especially surrounding the coated regions, with a minimally observed foreign body reaction but an expected presence of fibroblasts and, in some areas, granulation tissue with blood vessels within and around the implant. Most importantly, no signs of necrosis, apoptosis, infection, or excessive fibrosis were observed.

Helical devices implanted chronically were partially coated with polyethylene, which, as noted, was applied at the linear stage of fabrication to ensure incorporation into the helical structure. Polyethylene (a class of polyolefins) has been widely used as a catheter and cardiac lead coating [27]. However, some evidence exists to suggest that longer implant durations of polyethylene-coated cardiac leads can lead to signs of stress-cracking and severe oxidation that may affect the removability of these devices [28]. The collected removal forces did not suggest this effect; nonetheless, we still visually assessed the coatings of the devices. Shown are the coating-to-no coating transition point of a control device that was never implanted (Figure 6c, elongated for comparison) and two chronically implanted devices (Figure 6d, Platinum–Iridium with coating at 365 days; Figure 6e, Gold with coating at 461 days). Only minimal changes in material opacity were observed (cloudiness, possibly indicative of oxidative stress), and no stress fractures following chronic implantation and removal from rodents were noted.

The helical devices were also visually assessed for the presence of tissue fragments remaining on the device after removal. No pieces larger than 1 mm^3^ were observed on any removed device, and the remaining tissue that was observed on the removed devices were intermittent only. Generating histological slides from these areas proved difficult as those pieces were not securely adhered to the removed devices. The presence of remaining tissue on a device is pointed out by arrows in Figure 6e. Furthermore, following the removal of all devices studied here, we performed fluoroscopy to ensure there were no fractures or remaining fragments. As noted, we observed one fracture during the removal of a Gold helical device in the chronic rodent study. To further assess whether fragments remained after removal that may not be visualizable under fluoroscopy, we performed ICP-MS on the tissue samples (n = 8) following the removal of helical Platinum–Iridium devices of 2 cm in length (implanted subcutaneously for 60 days). Tissue samples that were analyzed for trace metals by ICP-MS contained on average of 1.99 ± 3.87 μg of Platinum (0.21 ± 0.41 μg of Iridium). The trace presence of residual Pt and Ir is indicative of dissolution rather than fragmentation given the consideration of the relatively large surface area of the removed microwire structure (~500 mm^2^) and represents less than 0.02% of the device by weight [29,30].

### 3.5. Proposed Mechanistic Models

**Figure 7:**
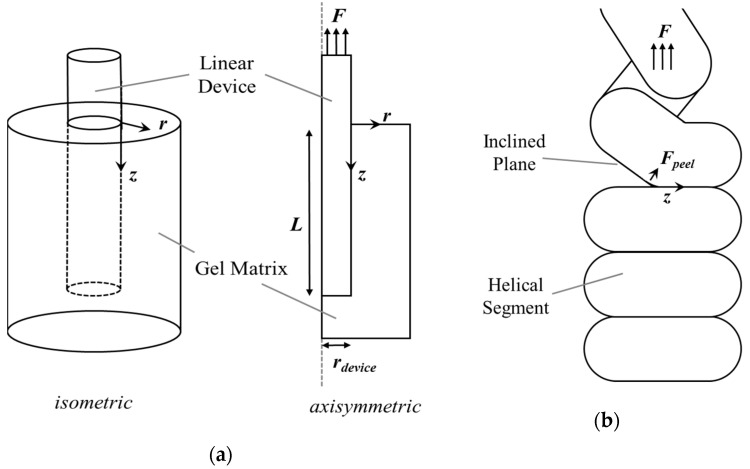
(**a**) A model of the removal of a linear device, which results in crack propagation along Z for a device of length L, the radius of the device, and a gel matrix radius of r. (**b**) A depiction of the hypothesized peel force, Fpeel, occurring during the removal of a helical device coil by coil as one helical segment separates from another and the gel at the inclined plane interface as the crack propagates along direction Z.

The removal of thin fibers from an elastic medium like synthetic gel or tissue has been well described by the shear lag model [31] and is dependent on variables such as the device radius, elastic modulus of the device and medium, radius of the medium, adhesion forces between the medium and the device, and length of the device. This is noted in the model depicted in Figure 7a, where direction Z indicates the direction of the crack propagation and debonding of the device from the medium during the removal of a linear device [32].

**Figure 8:**
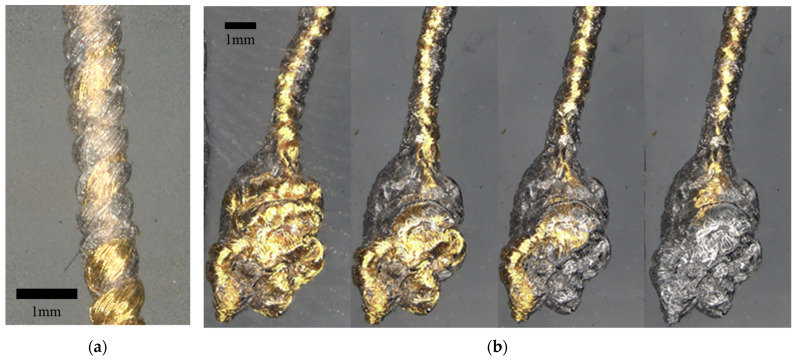
(**a**) A high-resolution image of the inward collapse of the helical device, leaving behind an imprint of the device in the gel. (**b**) A “bunched” non-linear placement of a helical device and the stepwise removal process.

No published model exists that describes the removal forces of a ductile helical device. Our observations suggest that as the helical wire structure device collapses inward during elongation, each coil individually pulls away from the surrounding tissue and into the space left by the hollow core, as shown in Figure 8a. This transmits removal force along an inclined plane (Figure 7b), resulting in adhesion separation via a peeling force, dramatically lowering the effect of removal on a given point of tissue as well as the helical device itself. In addition, the crack propagation time is itself elongated by traveling along Z horizontally at the interface of the helix and medium. Since the inter-coil spacing (pitch) is minimized in this design with each coil touching the next, the crack propagation is also continuous. Over the duration of removal, this lowers the chance of undesirable tissue “cutting” or the device failing or fracturing by lowering the maximally imparted force on the device.

## 4. Discussion

For compelling reasons, implantable neuromodulation device design should include removability as a core design input. Such a design input would necessitate an output focused on the maximal force required to remove devices in a clinically relevant setting. Here, we evaluated this removal force for a linear and helical design of a microwire structure electrode. Of importance were the method development steps initially taken to ensure consistent data collection. These included the development of a custom capstan gripper for accurate tensile testing, a vacuum oven treatment to tightly gel-encapsulate devices (without this treatment, removal forces were inconsistent and below 0.5 N for any device), and refinement of the surgical technique to locate the end of the devices under fluoroscopy and perform a minimally sized cut (<1 cm) to explant the device with surgical tools.

Initial devices were fabricated from Gold microwires due to their commercial availability as a commodity in the wire bonding industry for microchips. However, using Gold as a stimulation electrode in vivo presents electrochemical limitations. Gold electrodes tend to exhibit lower charge storage and charge injection capacities relative to Platinum and Iridium electrodes [33]. In addition, Gold is susceptible to dissolution under a high current density and high frequency stimulation conditions, particularly during the anodic phase of stimulation pulsing [34]. Platinum–Iridium has a tensile strength of 380 to 620 MPa [35], which is substantially greater than pure Gold at 100 to 220 MPa [36], and it is corroborated by the differences in ultimate tensile failure force for our devices at 32.9 versus 4.3 N. This difference was likely further exacerbated by the annealed state of the material, which is known to reduce tensile strength and standard in the bonding wire fabrication process [37]. The material difference was apparent across the chronic rodent removal forces from rodents, which were significantly different (*p* < 0.001) between Gold (1.82 ± 1.38) and Pt-Ir (3.76 ± 1.27) across all time points. However, the maximal removal forces from pigs (3.57 ± 1.71) for helical Pt-Ir devices were not different from rodents (*p* = 0.576). This suggests that the rodent serves as an acceptable vertebrate alternative to the larger pig species in the study of chronic removal forces of medical devices.

Percutaneous neuromodulation devices of helical design, which extend across the skin for direct electrical connections, are already used clinically [19,20]. The maximal removal forces for one of these devices has been reported to be no more than 3.2 N across 60 days of evaluation [38], while some lead fractures were still observed [8]. Of note is that these helical devices are only placed temporarily due to their percutaneous nature, and as such, the effects of chronic encapsulation on removal forces is less understood. Here, we evaluated the removal forces in a chronic biological model across a 1-year period and showed that the helical structure results in a chronically low force of removal independent of the base material (Platinum–Iridium or Gold). To achieve a chronic evaluation, the linear and helical devices used in this study needed to be fully implanted, which, unlike percutaneous helical devices, require the fluoroscopic finding of the device during surgical removal. This procedure would sometimes result in devices being interfaced at a point that was not the most distal end. However, for helical devices, we found that there was no difference in the removal force (*p* = 0.470) whether pulling was carried out from the middle (2.19 ± 0.20 N, n = 5) or end (2.41 ± 0.61 N, n = 5) of a device from a synthetic gel.

We note several limitations to the methods and results in this study. These include the lower number of samples evaluated at the 1-year mark and beyond (n = 7 total for both Gold and Platinum–Iridium helical devices) and the fact that large animal evaluations only extended to the 2-month mark. For the implantation of the devices, surgical placement limitations also needed to be accounted for, such as the placement of a device across muscle planes and joints which are likely to increase the risk of device failure, differing tissue mechanics that would alter removal forces, and the effect alternative surgical placement paths have on the ultimate removal force, which are all scenarios that could be further investigated. An analytical limitation includes the varied device lengths evaluated across this study, which was a result of continuous device development and refinement towards a single device length (or set of lengths). Another limitation included a lack of exploration on the effects different coatings may have on device removal forces. Lastly, we found that the injection of the helical structure can take a curvilinear path and can also be placed in bunched anchoring structures of overlapping paths by placing additional lengths of devices in a single location (without moving the needle). Preliminary results (Figure 8b) suggest that these “bunched” non-linear paths are removed in a first-in-last-out process that has a negligible effect on the removal forces for the helical device, and a future concerted investigation should aim to quantify any potential differences the removal path would have on maximal removal forces.

To the authors’ knowledge, this is the first report that has aimed to fill the gap in public knowledge regarding the removal forces experienced by a neuromodulation lead from both benchtop and animal models; it may help inform best practices for equivalent studies on the removal of other medical devices and neurostimulators. The benchtop setup addresses primary concerns in acquiring a valid result during the tensile testing of leads with the use of custom capstan grips. The benchtop and animal results were consistent with each other in regard to comparing helical and linear devices, providing fundamental data to be used in model development and validation. We also present, for the first time, data comparing the effect of structure on biologically relevant removal forces in a chronic animal model (107 days) and include data points following over 1 year of implantation, which has also not been previously reported elsewhere. These advancements in methods and their results support the novelty of the study presented here.

From the perspective of safety, the helical structure manufactured from Platinum–Iridium microwire was shown to provide the greatest overall safety factor (4.5×), and no adverse events (including fracture) or changes in ambulation were reported following the removal of this device variant in the subset of survival animals observed after device removal. Future work is aimed at evaluating the use of the Platinum–Iridium helical microwire structure electrode in eliciting stimulation effects on peripheral nerves, such as an efferent motor response, in rodent and porcine models while addressing the limitations encountered here. In conclusion, an open-core helical structure that can freely elongate appears to be a design that significantly reduces the force of removal both acutely and chronically. We envision this removal process to be achieved in future procedures using a needle-based technique to further reduce invasiveness and minimize the tissue trauma generally experienced during device removal procedures.

## 5. Patents

The work reported here is based on the following patented technologies:

- WO2022246007A1: System and methods for minimally invasive removal of implanted devices;
- WO2022182377A1: Injectable electrode with helical wire structure and methods for minimally invasive anchoring and removal;
- WO2021102195A1: Injectable wire structure electrode and related systems and methods for manufacturing, injecting and interfacing.

## Data Availability

The original contributions presented in this study are included in the article, and further inquiries and requests for data can be directed to the corresponding author.
